# Can lay health workers support the management of hypertension? Findings of a cluster randomised trial in South Africa

**DOI:** 10.1136/bmjgh-2017-000577

**Published:** 2018-02-15

**Authors:** Jane Goudge, Tobias Chirwa, Sandra Eldridge, Francesc Xavier F Gómez-Olivé, Chodziwadziwa Kabudula, Felix Limbani, Eustasius Musenge, Margaret Thorogood

**Affiliations:** 1 Centre for Health Policy, Faculty of Health Sciences, School of Public Health, University of the Witwatersrand, Johannesburg, South Africa; 2 Faculty of Health Sciences, School of Public Health, University of the Witwatersrand, Johannesburg, South Africa; 3 Centre for Primary Care and Public Health, Barts and the London School of Medicine and Dentistry, Queen Mary University of London, London, UK; 4 MRC/Wits Rural Public Health and Health Transitions Research Unit (Agincourt), Faculty of Health Sciences, School of Public Health, University of the Witwatersrand, Johannesburg, South Africa; 6 Division of Epidemiology and Biostatistics, Faculty of Health Sciences, School of Public Health, University of the Witwatersrand, Johannesburg, South Africa; 5 Statistics and Epidemiology Unit, Warwick Medical School, University of Warwick, Coventry, UK

**Keywords:** cluster randomised trial, South Africa, lay health workers, chronic care, hypertension

## Abstract

**Introduction:**

In low/middle-income countries with substantial HIV and tuberculosis epidemics, health services often neglect other highly prevalent chronic conditions, such as hypertension, which as a result are poorly managed. This paper reports on a study to assess the effect on hypertension management of lay health workers (LHW) working in South African rural primary healthcare clinics to support the provision of integrated chronic care.

**Methods:**

A pragmatic cluster randomised trial with a process evaluation in eight rural clinics assessed the effect of adding two LHWs supporting nurses in providing chronic disease care in each intervention clinic over 18 months. Control clinics continued with usual care. The main outcome measure was the change in the difference of percentage of clinic users who had elevated cardiovascular risk associated with high blood pressure (BP) before and after the intervention, as measured by two cross-sectional population surveys.

**Results:**

There was no improvement in BP control among users of intervention clinics as compared with control clinics. However, the LHWs improved clinic functioning, including overall attendance, and attendance on the correct day. All clinics faced numerous challenges, including rapidly increasing number of users of chronic care, unreliable BP machines and cuffs, intermittent drug shortages and insufficient space.

**Conclusion:**

LHWs improved the process of providing care but improved BP control required improved clinical care by nurses which was compromised by large and increasing numbers of patients, the dominance of the vertically funded HIV programme and the poor standards of equipment in clinics.

**Trial registration number:**

ISRCTN12128227.

**Key questions**

**What is already known about this topic?**

- Systematic reviews have provided evidence of the effectiveness of lay health workers (LHW) in improving access to care, the quality of care, including screening for cardiovascular risk factors, reducing systolic blood pressure (BP), fasting blood glucose and weight, as part of community-based care.
- While there is systematic review of studies looking at the effect of task shifting, the focus was shifting prescribing from doctors to nurses, we found no trials looking at LHWs’ role in the provision of clinic-based integrated chronic care.

**What are the new findings?**

- The Nkateko study was the first randomised controlled trial to assess the role of LHWs in the provision of integrated chronic care.
- While we found no improvement in BP control, the LHWs improved clinic attendance.
- A large and increasing numbers of patients, the dominance of the vertically funded HIV programme and the poor standards of equipment in clinics compromised the quality of clinical care provided by nurses.
- Assistance from LHWs with booking appointments, sending reminders, prepacking medication and providing health education was insufficient to improve BP control in this environment.

**Recommendations for policy**

- Our results, taken together with the existing evidence, suggest that LHWs can play an important role in supporting the provision of integrated chronic care.
- However, adding additional human resources (even if readily available and relatively inexpensive) is unlikely to have an effect on health outcomes, without the necessary equipment to accurately measure BP, and sufficient clinical staff to treat the growing numbers of chronic patients.

## Introduction

Low/middle-income countries are facing an increasing burden of chronic non-communicable diseases, including a high prevalence of hypertension.^1^ In countries with high levels of HIV and tuberculosis (TB), the result is a considerable burden of care in primary healthcare clinics. In South Africa, the health service is managing the world’s largest HIV treatment programme,^2^ but hypertension, which is more prevalent than HIV, is poorly managed with low levels of awareness and control.^3 4^ In the Agincourt Health and Demographic Surveillance System (HDSS) site, based in rural north-east South Africa, around 57% of the adult population have high blood pressure (BP), but the condition is appropriately managed in less than 10%.^5^

Primary healthcare clinics in South Africa are responsible for case finding, treatment and adherence support for patients with chronic diseases. In an effort to provide integrated care for the large number of patients with chronic disease, services have recently been reorganised with appointment scheduling, preappointment retrieval of files and preparing medication prior to the patient’s appointment.^6–8^ The aim is to speed the journey for chronic patients through the clinic, improving efficiency, while ensuring chronic conditions are effectively controlled. A recently published review of these changes showed that nurses were struggling to carry out these tasks in addition to clinic consultations.^9^

Task shifting has been an important strategy in the provision of care for patients with HIV and TB.^10^ Lay counsellors provide counselling and testing services,^11–14^ and lay health workers (LHW) provide adherence support.^15–17^ We hypothesised that LHWs would be able to assist clinic staff with the administrative and education aspects of the newly reorganised integrated chronic care, allowing the nurses to focus on the clinical consultations and hence leading to improve health outcomes. In this paper, we report the results of a parallel cluster randomised trial which tested a clinic-level intervention, shifting tasks from nurses to LHWs. We aimed to improve the management of chronic conditions, specifically the management of hypertension.

## Methods

### Study setting

The trial was based in Bushbuckridge subdistrict in Mpumalanga Province, South Africa, where the MRC/Wits Rural Public Health and Health Transitions Research Unit has been running the Agincourt HDSS since 1992.^18^ The Agincourt HDSS, covering an area of 450 km^2^, includes 115 000 individuals living in approximately 20 000 households distributed in 32 villages. The area has high unemployment with more than 50% of men (aged between 25 and 54 years old) and more than 20% of women (25–49 years old) migrating to urban areas for work for part of the year.^19^ Infrastructure has improved in the last few years, but there is still irregular water supply, electricity is unaffordable for many, and the schools and health facilities do not provide services of adequate quality.^18^

Ten primary healthcare facilities and three hospitals that are 25–60 km away from the site serve the local population. The Department of Health piloted the provision of integrated chronic disease care (ICDM) in the Bushbuckridge subdistrict, 2 years prior to the start of this study. It has since been rolled out to other provinces.

### Study design, randomisation and blinding

**Figure 1:**
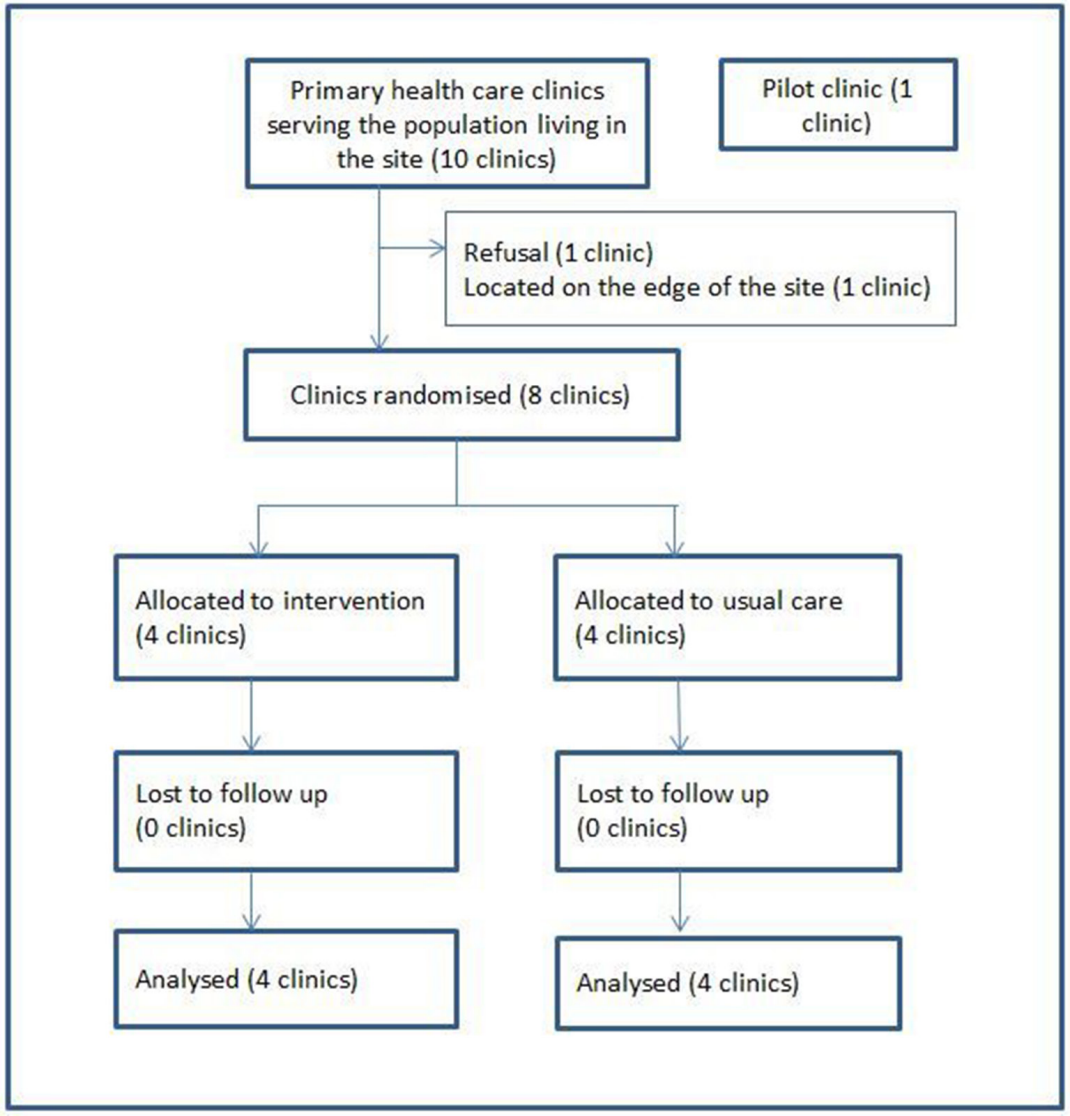
Allocation of clinics.

The study was a pragmatic cluster randomised controlled trial with repeated cross-sectional surveys.^20 21^ Each cluster was one clinic together with the population that it served. Clinics were included if they were located in the site, rather than on the periphery, and if the clinic manager consented to participate in the study (figure 1). A clinic located out of the study site was chosen as a pilot site, to learn lessons about implementing the intervention prior to establishing it in the trial sites. Simple 1:1 random allocation of the eight clinics was carried out at a public meeting in the presence of clinic staff and community members. Clinics’ names in sealed envelopes were drawn from a box by a community member. This transparent process facilitated understanding and trust in the randomisation. It was not possible to blind clinic staff or patients to the allocation. However, the primary outcome was measured using an encrypted data set, with no indication of which arm received the intervention.

### Usual care in the study site

On attending a clinic, each patient is assigned to one of two queues, one for patients with an ongoing chronic condition, another for the remaining patients. After obtaining his/her file, each patient has their temperature, pulse, weight and BP measured at a ‘vital signs’ station. This is usually carried out by a junior (enrolled) nurse, before the patient is seen by a professional nurse, who diagnoses, prescribes and dispenses medication in a designated chronic care consultation room. The operation of the separate chronic disease pathway depends on sufficient staff and equipment to maintain two vital signs stations (one for each queue), to carry out the additional tasks (preappointment retrieval of files, appointment scheduling and predispensing of medication), and that patients come within a few days of their appointed day, otherwise prepacked medication has to be unpacked.

### Intervention

The intervention consisted of two LHWs assigned to each clinic to support nurses in the management of patients with chronic disease. The LHWs, selected from the local community, had completed their secondary education but had had little further education. They were trained and supervised by an implementation manager, who was a local primary healthcare nurse, with a training qualification. As part of the pragmatic design, the staff and supervisors of intervention clinics were able to decide which tasks the LHWs should do. All the intervention clinics chose to have the LHWs assisting with booking appointments, retrieving and filing patient files, and providing health education (on adherence and lifestyle). They also all chose to have the LHWs taking measurements in the vital signs queue and assisting the nurses with the prepacking of medications. Some clinics struggled to find space for these activities to take place, so the LHWs often had to operate in the corridor or the reception area. In addition, the LHWs phoned or sent a text message to patients with hypertension to remind them of their appointment. The implementation manager ensured fidelity to the planned intervention. The four control clinics continued working as normal and the implementation manager did not visit them or interact with their staff to avoid contamination.

In the first months of the intervention, the implementation manager found that none of the intervention clinics had cuffs in good repair for the electronic BP machines. Without the possibility of correctly measuring BP the intervention had little chance to be effective. Thus, although this was a pragmatic trial, we decided that it was necessary to replace the cuffs. To keep the comparison meaningful, we provided both the intervention and control clinics with two new sets of cuffs. We originally planned the intervention to last for 15 months, but after having replaced the cuffs, we extended the intervention period for a further 3 months, up to 18 months in total.

### Primary and secondary outcomes

Our primary outcome was based on the South African guidelines for the management of hypertension, which call for a focus on people at moderate or greater cardiovascular risk.^22^ The primary outcome is defined as the difference (between intervention and control clinics) in the change of the proportion of the population who have uncontrolled hypertension together with a risk profile indicating at least moderate risk of cardiovascular disease (CVD).^20^ We did not have the resources to make the clinical diagnoses called for in the guidelines and used proxy measures for some items. For example, we collected data on self-reported stroke rather than making a diagnosis, and we used waist circumference as a proxy measure of obesity. The participants whose data were used to calculate the outcome measures were the self-defined users of one of the eight clinics. As there were no records of who attended a clinic, and individuals are free to attend any clinic they choose, we asked respondents which was their usual clinic and which clinic they visited last. For the large majority of respondents the usual clinic was the same as the last clinic. We used the usual clinic for the analysis of the primary outcome.

Our secondary outcomes are listed in box 1. The first five were derived from the two population surveys, while the last two came from the data we collected from patients as they entered the clinics, and from their clinic files. Data for the fifth outcome were not collected, as the time frame of 1 year was omitted from the survey questionnaire. There was some delay in setting up the system for collecting clinic data; as a result, the last two outcomes are reported for the period May 2014 to July 2015.

Box 1 **Secondary outcomes of the trial as reported in this paper**

**Secondary outcomes derived from population surveys**

1. Changes in the proportion of the population at different levels of blood pressure-related cardiovascular risk by age group and sex.
2. Change in proportion of the population with undiagnosed hypertension.
3. Change in the proportion of the population reporting they had had their blood pressure measured.
4. Change in the proportion of the population reporting that they are using medication for hypertension.
5. Change in the proportion of people in the population reporting that they have attended a clinic in the last year. *Listed in the analysis plan but not collected.*

**Secondary outcomes derived from clinic activity data**

1. Retention in care of people with diagnosed hypertension defined by the proportion of appointments kept during the study period.
2. The number of clinic visits per month related to a diagnosis of hypertension. *Not listed in the analysis plan but used to replace secondary outcome no. 5 above.*

### Baseline and end of intervention cross-sectional surveys

**Figure 2:**
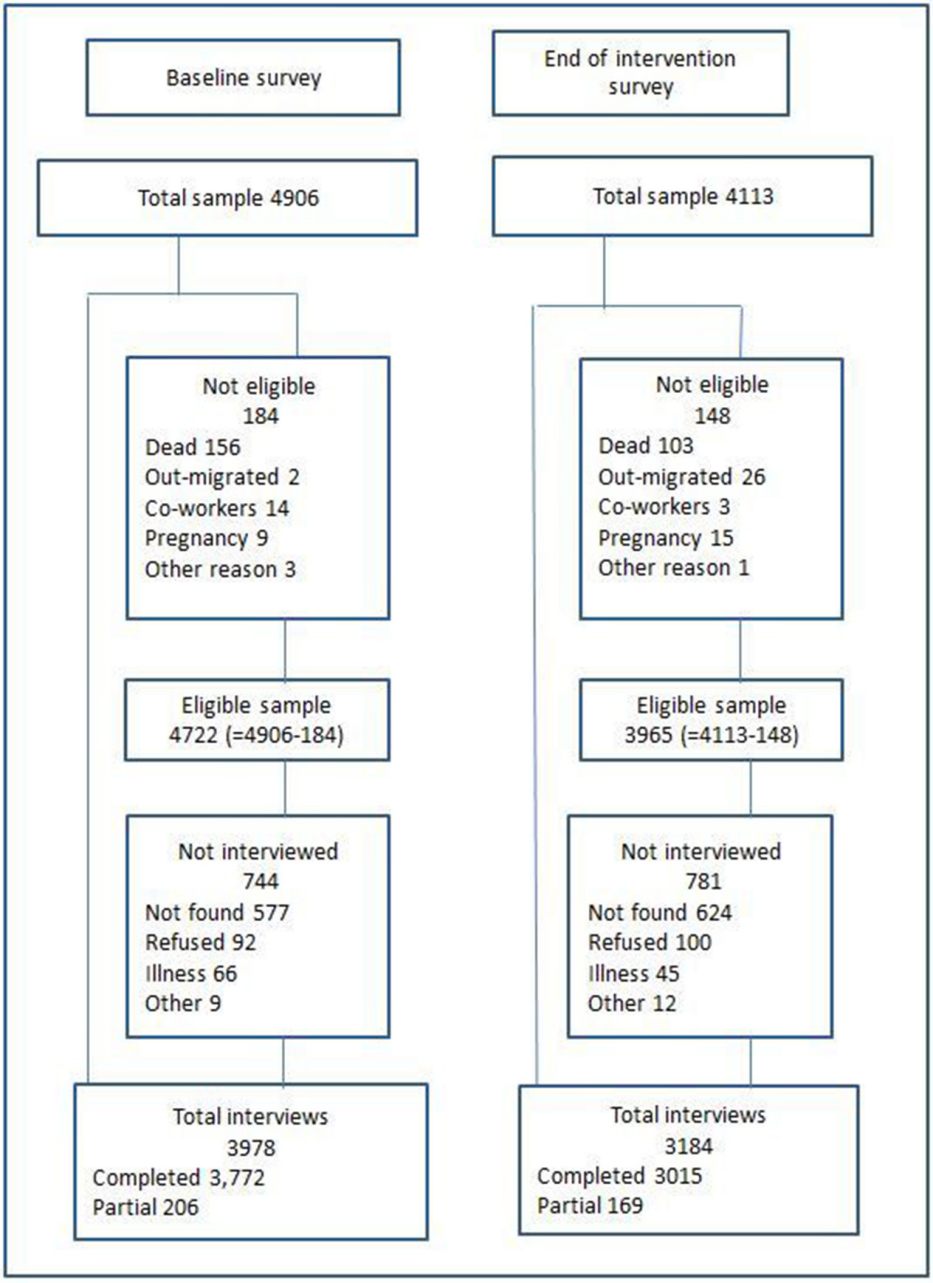
Response to survey by individuals sampled.

For the two surveys, a separate random weighted sample of people over 18 years was drawn from the Agincourt census database with no knowledge of which, if any, clinic individuals used. In the first survey, no other eligibility criterion was used. In the second survey, we excluded from the sampling frame individuals who had recently been randomly selected to participate in another research study that demanded a lot of participants’ time. The sample was weighted to provide larger numbers of older people, who could be expected to have a higher prevalence of hypertension. Informed consent was sought from each participant and refusals are reported in figure 2.

Fieldworkers were trained for 2 weeks before the survey. As the fieldworkers lived in the community it was not possible to blind them to which clinics received the intervention. However, they had no involvement in either the implementation or the process evaluation. The questionnaire was piloted in advance. Quality control included three stages in the first survey: a supervisor checked the completed questionnaires in the field; the project manager checked them as he filed them in the office; and the data manager checked them prior to data entry. In the second survey, we included an additional check of a random 5% of interviews for which a supervisor returned to the household a few days later and reinterviewed the participant to confirm responses to some selected questions. A double entry data system was used and a data manager made the comparison of the two entries and corrected those where there was a discrepancy using the paper questionnaire as a reference.

The questionnaire included self-reported history of hypertension, diabetes, stroke, heart failure, angina and heart attacks, smoking, clinic use (last and usual) and family history of CVD. We did not attempt to measure the number of cigarettes smoked as regular heavy smoking is unusual in this cash-poor community. Information on age, gender, marital status, education and socioeconomic status was drawn from the census database.

### Variable measurement and definitions

Pulse, systolic and diastolic BP was measured three times in a seated position after 5 min of rest and with 2 min interval using the OMRON M6W automated cuff (Omron, Kyoto, Japan). Waist circumference was measured using a flexible tape measure (SECA). A finger prick was used to measure random blood glucose (CareSens N Monitor) and total cholesterol (CardioChek PA Silver version).

We derived the mean systolic and diastolic BP using the average of the second and third BP measurements. We defined diabetes as a random glucose measure of over 11 mmol/L. A person with a random glucose measure between 7 and 11 mmol/L, and who reported that they had not eaten for 12 hours, was recorded as having high blood glucose. The remaining respondents were considered not to be diabetic. We defined obesity as a waist circumference greater than 94 cm in men or greater than 80 cm in women.^22^

We estimated the time that patients spent in the clinics by calculating the difference between the time the patients reported arriving at the clinics and the time of the exit interviews, as described under the Process evaluation section.

### Clinic activity data

To collect data on clinic activity, an experienced data entry clerk sat with a laptop in each control and intervention clinic for the period of the intervention. A unique record using identifiers (ID numbers, cellphone, date of birth, gender, village of residence and the name of another person in the household) was created for each consenting individual, and the date of visit, diagnosis and date of return visit were then collected from the clinic records for this and all subsequent visits by that individual.^23^

### Sample size

We derived our assumptions for the sample size calculation from data collected in the same site in 2010.^24^ We adopted the use of the coefficient of variation (SD of the cluster means divided by the overall mean) as used in similar study settings when we cannot get a good intracluster variation^25–27^ and assumed that the coefficient of variation would be similar in the two groups and that effects of the interventions would be similar across clusters. We used a background prevalence of 36% and a coefficient of variation of 0.132 (95% CI 0.087 to 0.177). We assumed that the two population surveys would each include at least 4000 participants, with approximately 500 people in each of the eight clusters. We estimated that we would have 88% power to detect a reduction of 11% (from 36% to 25%) in people at moderate or greater cardiovascular risk.

### Statistical methods

Sociodemographic and clinical information including both primary and secondary outcomes was summarised using frequencies and summary measures. Continuous variables such as age and BP levels were summarised using the mean and SD. Categorical variables such as gender were described through frequency tabulations by reporting the relative percentage and the number of observations (%, n).

The analysis of the primary outcome was conducted to test the difference in the change in the proportions with moderate or greater added risk of CVD in the intervention and control clusters. We used the cluster adjusted Pearson’s χ^2^ to adjust for clustering for each group. This analysis was carried out in EXCEL with user written commands. All other analyses were done using STATA V.14 (StataCorp, 2015. Stata Statistical Software: Release 14. College Station, TX: StataCorp) and statistical significance was considered at 5% level.

We included the following sensitivity analyses for the primary outcome: (A) an analysis which included those who did not name any specific drugs when reporting on hypertensive drugs (in the primary analysis these individuals were not included as having hypertension); (B) an analysis using the ‘last’ clinic rather than the ‘usual’ clinic to assign individuals to clinics; (C) an analysis using two-stage regression modelling instead of the adjusted χ^2^; and (D) an analysis using mixed effects model adjusting for covariates. Secondary outcomes were analysed in the same way as the primary outcome. No sensitivity analyses were conducted.

### Process evaluation

We conducted a theory-driven, mixed methods evaluation to understand the causal processes that led to change (or not).^20^ We observed clinic activity, the movement of patients along the clinic pathway and patient consultations over a period of 3–9 days at 6-month intervals (control and intervention) during the 18-month intervention period. We conducted brief exit interviews with the patients as they left the clinic (n=703). We also conducted bimonthly interviews with the LHWs throughout the intervention, as well as interviews with clinic staff, and clinic and district managers.

The clinic managers of all the clinics taking part in the trial consented for their clinic staff to participate and all individuals interviewed in the population surveys or process evaluation gave written informed consent to the interview.

### Role of funding source

The funder had no involvement in the study.

## Results

The baseline population survey collected information on 3978 people, with a response rate of 84.2% (3978/4722) (figure 2). After excluding 44 questionnaires that were completed by a fieldworker who was later found to be unreliable, 145 respondents who reported that they did not use any public clinic and a further 376 respondents who used a clinic that was not one of the trial clinics, 3413 questionnaires were included in the analysis. The end of intervention survey had a response rate of 80.3% (3184/3965) (figure 2). After excluding those who reported that they did not use public clinics (97) and those who did not use one of the trial clinics (548), 2539 questionnaires were included in the analysis.

There were no important differences in sociodemographic or health variables between the control and intervention groups (table 1). Just under half of the respondents were found to have hypertension. In 6%–10% the hypertension was controlled on treatment, 9%–13% were on treatment but the hypertension was not controlled, and between 20% and 30% of the respondents had hypertension but were not on treatment. As is common in the black South African population, obesity was more prevalent in women than men. There are fewer men aged over 80 years in the second survey because we excluded individuals who had just participated in another research study. This resulted in a very small sampling frame for this small group of men, and so we selected for interview all the available men.

**Table 1:** Sociodemographic and health variables in the baseline and end of intervention surveys

	Control		Intervention	
	Baseline survey n=1908	End of intervention survey n=1430	Baseline survey n=1505	End of intervention survey n=1109
Mean age (SD)	56.4 (19.8)	52.7 (19.7)	56.8 (18.9)	53.0 (19.2)
	**% (n)**	**% (n)**	**% (n)**	**% (n)**
Female	56.0 (1068)	70.5 (1008)	55.1 (829)	68.7 (762)
In a marital union	48.2 (919)	39.5 (565)	49.0 (737)	40.1 (445)
Education				
No education	40.6 (772)	37.2 (530)	38.4 (576)	32.3 (357)
Primary	25.4 (483)	21.7 (310)	25.4 (381)	21.9 (242)
Secondary	30.9 (588)	38.6 (550)	31.9 (479)	40.7 (451)
Tertiary	3.1 (58)	2.5 (36)	4.4 (66)	5.2 (57)
Socioeconomic status				
1 (lowest)	18.8 (326)	23.7 (335)	16.6 (229)	14.8 (164)
2	20.1 (348)	20.7 (292)	18.3 (253)	18.9 (209)
3	19.2 (332)	18 (254)	19 (263)	20.7 (229)
4	19.4 (335)	19.6 (277)	20.8 (288)	20.8 (230)
5 (highest)	22.5 (390)	18.1 (256)	25.4 (351)	24.7 (273)
Smoking history				
Never smoked	79.3 (1507)	84.9 (1214)	76.8 (1151)	85.8 (952)
Previous smoker	11.3 (214)	5.6 (80)	13.4 (201)	7 (78)
Smokes <1/day	2.3 (44)	2 (29)	2.4 (36)	1.6 (18)
Smokes >1/day	7.2 (136)	7.5 (107)	7.4 (111)	5.5 (61)
Self-reported health and risk				
Family history of CVD	8.3 (158)	10.4 (148)	7.4 (111)	6.1 (68)
Diabetes	6.5 (124)	6.9 (99)	6.2 (93)	5.8 (64)
Coronary heart disease	4.2 (81)	1.6 (23)	1.9 (29)	2.5 (28)
Stroke or TIA	3.2 (61)	3.2 (46)	2.6 (39)	1.6 (18)
Heart failure	2.8 (53)	1.5 (21)	2.4 (36)	1.2 (13)
Obesity				
Male (waist >94 cm)	26.3 (221)	20.4 (86)	31.1 (210)	30.3 (105)
Female (waist >80 cm)	80.2 (857)	78.5 (791)	81.4 (675)	77.3 (589)
Hypertension				
No hypertension	53.7 (1024)	52.9 (757)	53.0 (797)	50.9 (564)
On treatment and controlled	10.2 (194)	11.2 (160)	6.6 (100)	11.3 (125)
On treatment but not controlled	9.2 (175)	13.2 (189)	8.8 (133)	13.0 (144)
Not on treatment	27.0 (515)	22.7 (324)	31.6 (475)	24.9 (276)
Blood glucose				
Normal <11, not fasting	91.5 (1745)	92.7 (1325)	91.4 (1375)	92.1 (1021)
High 7 <11, fasting	0.7 (13)	0.9 (13)	0.3 (4)	0.8 (9)
Diabetic 11 or more	7.8 (149)	6.4 (92)	8.4 (126)	7.1 (79)

CVD, cardiovascular disease; TIA, transient ischaemic attack.

When we planned the study, we were concerned that many individuals might move between intervention and control clinics and so we asked respondents which was their usual clinic and which was the last clinic they used. We found that less than 3.5% of individuals had switched between intervention and control clinics (see online supplementary table 1).

10.1136/bmjgh-2017-000577.supp1 Supplementary file 1

### Primary and secondary outcomes

**Figure 3:**
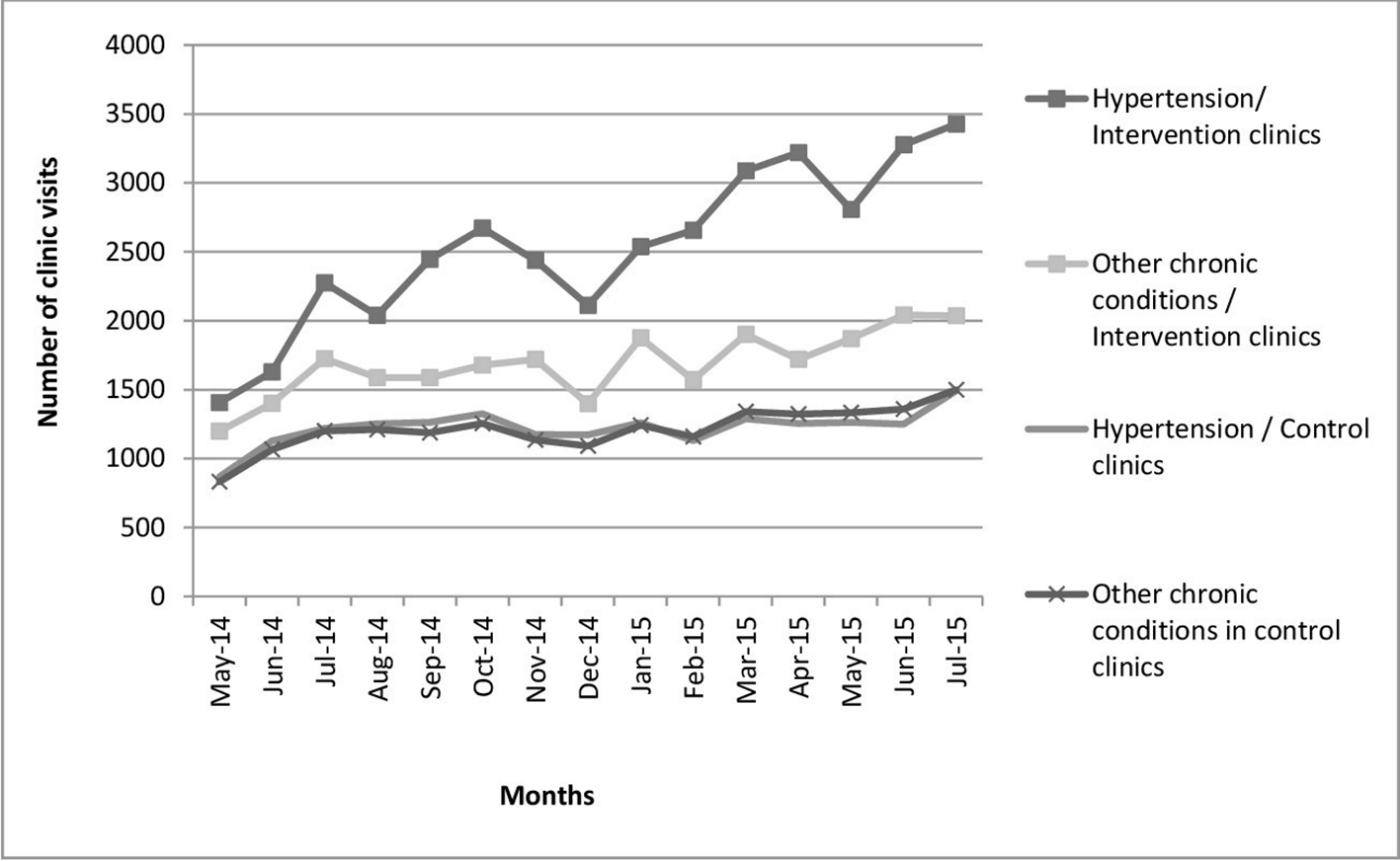
Number of monthly clinic visits by patients with hypertension and other chronic conditions in the control and intervention clinics.

There was no evidence of an effect of the intervention in the primary outcome or in the first four secondary outcomes that were derived from the population surveys (table 2). None of the sensitivity analyses altered this conclusion. There was no evidence of a reduction in those with moderate or greater CVD risk (OR 1.13, 95% CI 0.83 to 1.54). The 95% CI excluded the OR of 0.59 given by the clinically important difference in our sample size calculation, providing very strong evidence that the intervention as delivered could not have achieved the desired change. By the end of the intervention, three quarters of patients with hypertension were attending the intervention clinics on the day of their appointment, compared with 56% in control clinics (figure 3), suggesting improved adherence.

**Table 2:** Comparison of primary and secondary outcomes at end of intervention by study arm

	Control n=1414	Intervention n=1094	Estimated ICC	Adjusted OR (95% CI)	Adjusted χ^2^ statistics	P value
	% (n)	% (n)				
CVD risk						
No or low	74.1 (1048)	73.2 (801)	0.006	1.13 (0.83 to 1.54)	0.076	0.782
Moderate or higher	25.9 (366)	26.8 (293)				
Gender						
Female						
No or low	74.2 (742)	74.8 (564)	0.004	1.00 (0.75 to 1.34)	0.039	0.844
Moderate or higher	25.8 (258)	25.2 (190)				
Male						
No or low	73.9 (306)	69.7 (237)	0.013	1.32 (0.85 to 2.04)	0.59	0.443
Moderate or higher	26.1 (108)	30.3 (103)				
Age						
18–29 years						
No or low	99.0 (188)	95.9 (117)	0.183	22.5 (0.122 to 4.144)	0.3	0.584
Moderate or higher	1.1 (2)	4.1 (5)				
30–39 years						
No or low	90 (206)	89.6 (173)	<0.001	1.04 (0.55 to 1.95)	0.012	0.914
Moderate or higher	10 (23)	10.4 (20)				
40–49 years						
No or low	87.2 (224)	84.8 (167)	<0.001	1.22 (0.72 to 2.08)	0.532	0.466
Moderate or higher	12.8 (33)	15.2 (30)				
50–59 years						
No or low	75.1 (148)	77.3 (136)	0.004	0.88 (0.50 to 1.54)	0.186	0.666
Moderate or higher	24.9 (49)	22.7 (40)				
60–69 years						
No or low	67.3 (142)	68.0 (106)	0.012	0.97 (0.62 to 1.51)	0.01	0.92
Moderate or higher	32.7 (69)	32.0 (50)				
70–79 years						
No or low	44.1 (82)	47.4 (64)	0.037	0.88 (0.43 to 1.77)	0.125	0.723
Moderate or higher	55.9 (104)	52.6 (71)				
80+ years						
No or low	40.3 (58)	33.0 (38)	<0.001	1.37 (0.82 to 2.28)	1.435	0.231
Moderate or higher	59.7 (86)	67.0 (77)				
Hypertension diagnosis						
Undiagnosed	22.1 (312)	24.1 (264)		1.12 (0.93 to 1.35)		
None or diagnosed	77.9 (1102)	75.9 (830)	<0.001		1.489	0.222
BP ever measured						
No	5.7 (80)	9.1 (100)	0.014	0.71 (0.29 to 1.73)	1.729	0.188
Yes	94.3 (1334)	90.7 (994)				
Taking medication for hypertension						
No	75.6 (1069)	75.7 (828)	0.024	1.21 (0.70 to 2.09)	0.002	0.988
Yes	24.4 (345)	24.3 (266)				

BP, blood pressure; CVD, cardiovascular disease; ICC, intraclass correlation.

**Figure 4:**
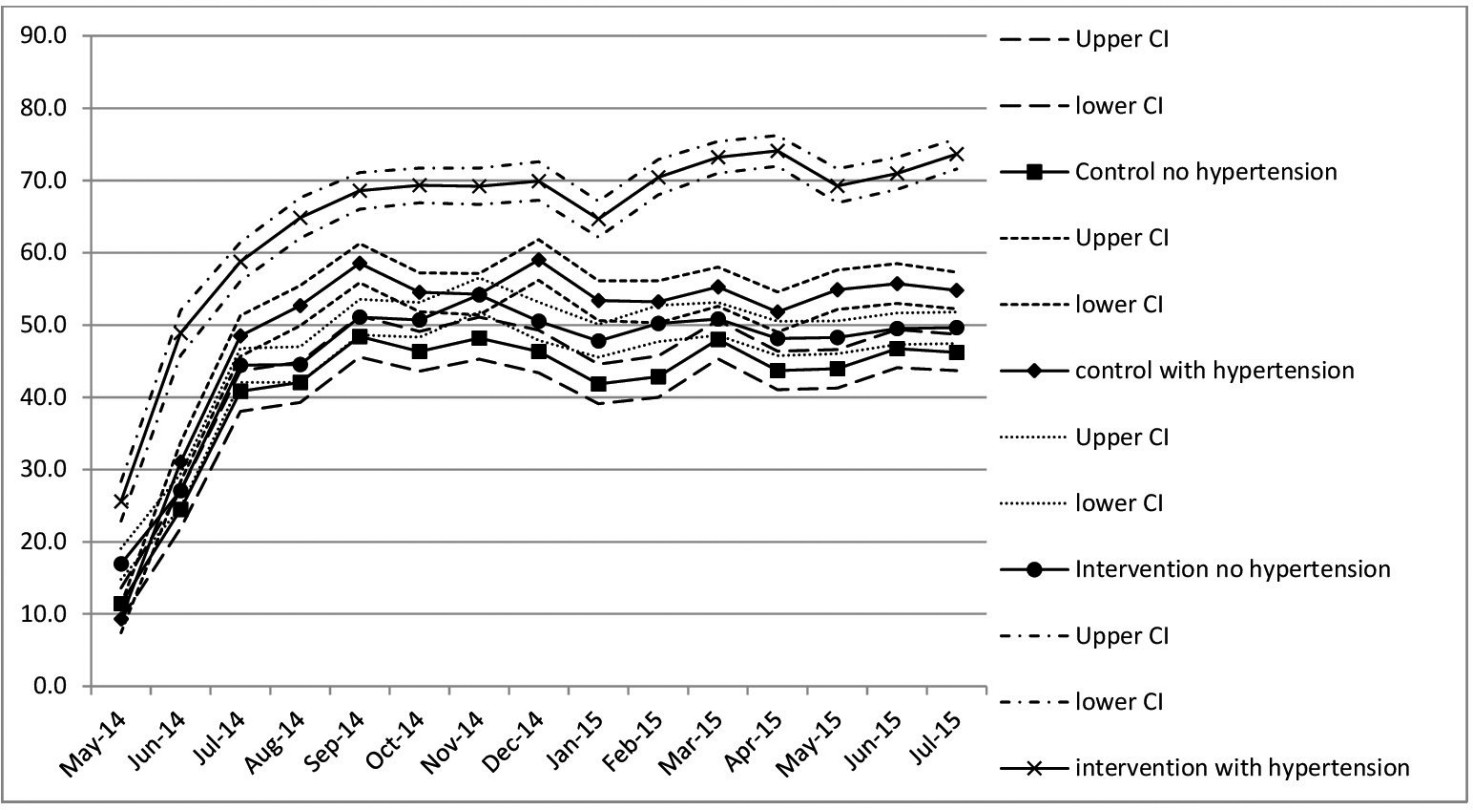
Percentage of patients who attend the clinic on their appointed day by month.

All clinics experienced an increase in the number of visits by patients with a chronic condition (figure 4) (analysis of variance coefficient 53.3; P<0.01). The number of visits in control clinics was similar for those with and without hypertension. However, in the intervention clinics the number of visits by chronic patients (with and without hypertension) was greater than in the control clinics (figure 4). In particular, there were a significantly greater number of hypertension visits than other chronic condition visits in the intervention clinics (analysis of variance for patient with hypertension coefficient 1295; P<0.01, chronic conditions coefficient 463; P<0.01).

### Process evaluation

The almost doubling of the number of patients with a chronic disease attending the clinics over the 18 months of the study, with no matching increase in equipment or space, resulted in both control and intervention clinics facing numerous challenges. These included BP machines that often failed to function, worn out cuffs, intermittent shortages of drugs and insufficient space for the increasing numbers of patients. The cuffs that we replaced early on in the trial were worn out before the end of the intervention. There was no routine servicing of the BP machines, and when they were taken for repair, they could be away from the clinic for several months. Sometimes a clinic only had functioning mercury sphygmomanometers, which the nurses were reluctant to use because of the time involved and the discomfort of using stethoscopes for several hours at a time. Table 3 provides a description of the state of the BP machines for different periods of the study.

**Table 3:** Description of the state of the BP machines from observations and interviews

Study period	Intervention clinics	Control clinics
January to June 2014	Two clinics stopped using electronic machines due to faulty cuffs and used manual machines. In one clinic the machine did not function properly and readings were unreliable.	Generally all electronic BP machines were functioning well. Although all cuffs were wearing out.
	Cuffs for electronic machines provided to all clinics	
July 2014 to March 2015	Cuffs for manual machines were still a problem. Electronic machines themselves began to develop problems as a result of overuse.	Little information in this period
April 2015 to August 2015	Problems with the electronic BP machines increased (with the on/off button not working, or the machine not clearing the data in preparation for the next patient). At the end of the period three of the four intervention clinics had their machines sent for repair. Clinic managers had little hope of getting them back soon. Cuffs supplied by study had started wearing out.	Early in this phase one clinic sent its machines for servicing, but it was not returned by the end of study. Two clinics doubted the accuracy of readings and used a manual machine to confirm a high reading. Cuffs supplied by study had started wearing out.

BP, blood pressure.

Despite these challenges, there were signs of improvement in the provision of care. The LHWs were encouraged to support the nurses in identifying undiagnosed hypertension among patients attending for other reasons. In the intervention clinics, 760 patients without a diagnosis of hypertension were identified with high BP and half of them (40%, 301 individuals) subsequently received a diagnosis of hypertension during the 18 months. Data from patient exit interviews suggest that waiting times may have been reduced in the intervention clinics (table 4).

**Table 4:** Mean time in hours spent by patients with hypertension in clinics derived from exit interviews

	Control			Intervention		
	Phase 1 March to July 2014	Phase 2 November to March 2015	Phase 3 June to August 2015	Phase 1 March to July 2014	Phase 2 November to March 2015	Phase 3 June to August 2015
Mean (SD)	3.67 (1.77)	3.96 (1.17)	3.60 (1.29)	3.30 (1.22)	3.07 (0.99)	2.41 (1.16)
n	49	105	95	126	88	95

## Discussion

We aimed to improve the management of hypertension by providing extra support from LHWs to the nurses who are responsible for the management of chronic conditions in primary care clinics. We found no evidence of an improvement in the management of hypertension as a result of our intervention. However, we did find improvements in the functioning of the intervention clinics in a number of important ways, including patients keeping their appointments, and more patients with hypertension returning to the clinics.

This was a pragmatic trial, designed to test an intervention that was both affordable and sustainable in a sub-Saharan Africa context. Using the precis-2 tool, we (JG, MT) assessed that the trial had a score of 41 out of 45, where 45 is the most pragmatic.^28^ We aimed to only provide those parts of an intervention which, if effective, could conceivably be funded by the South African Health budget. We provided some training for the nurses, and the implementation manager tried to assist in getting the BP machines repaired using the normal systems. However, we did not supply extra BP machines. We did supply replacement cuffs for existing machines, but supplied these to both intervention and control clinics. Moreover, in keeping with the pragmatic nature of the trial, and to encourage clinic staff to feel ownership of the intervention, the ways in which the LHWs would support chronic disease care were decided separately in each clinic by the clinic staff.

The trial was based on a strong research platform provided by the HDSS which allowed us to carry out stratified random sample surveys and also to collect information on clinic activity. The population surveys, which measured the BP control of respondents who usually used the study clinics, were designed to identify a change in the numbers accessing treatment for hypertension as well as a change in BP control. However, this measure included those who might not have attending the clinic as regularly as they should have been, reducing our chances of identifying the effect of improved care on BP control among those who attended a clinic regularly. (Alternative study designs were not possible: existing clinic records were not sufficiently reliable; intervening to strengthen the current filing system would have reduced the pragmatic nature of the trial; and setting up a study measuring station for each clinic was not affordable.) Moreover, as there were only eight clinics in the HDSS we were limited in the number of possible clusters.

Through the implementation manager we were able to ensure fidelity to the planned intervention as well as sufficient flexibility to the local context. We were also careful to separate implementation and evaluation activities. It is possible that the activity of the trial contributed to improved performance in the control clinics. We had a data entry clerk in all the control clinics who collected information about clinic users and, inevitably, contributed to the management of the clinic files, and therefore relieved nurses of some routine tasks. However, we do not think this possible contamination was sufficient to reduce a difference between control and intervention outcomes.

The lack of effect in BP control may be due to the poor condition of the BP machines and cuffs, and that both the intervention and the control clinics were overwhelmed by a rapid increase in numbers of chronic patients. This increase was due both to the continuing roll-out of antiretrovirals for HIV and a hospital policy of referring outpatients back into primary care. In the intervention clinics, there was a further increase in patients with hypertension attending the clinic, most probably due to the intervention itself.

An earlier study of the ICDM initiative in the same clinics demonstrated that management of HIV was more effective than management of BP: less than 50% of the patients attending the clinic had controlled BP, nearly 90% of patients with HIV had a controlled CD4 count.^9 28 29^ South Africa’s HIV programme is vertically organised, with generous funding and a separate management structure, requiring more detailed reporting from primary care clinics. It may be that this also continued to distort the work in the clinics, despite attempts to integrate the provision of care; with no requirement to report patient outcomes for hypertension there is insufficient emphasis on improving clinical care.^30^

Systematic reviews of randomised trials have concluded that community health workers are effective in improving health and treatment outcomes,^31^ the included studies predominately focus on a single condition or health outcome (breast feeding, immunisation, TB cure rates), rather than the provision of integrated care. Moreover, the health outcomes measured are often those achieved by the community health worker alone, rather than outcomes requiring the contribution of other healthcare workers, as in this study. We found only one systematic review of studies looking at the effect of task shifting, which focused only on shifting prescribing from doctors to nurses.^32^ As far as we are aware, the Nkateko study was the first randomised controlled trial to assess the supportive role of LHWs in the management of hypertension in the context of clinic-based integrated chronic care.

## Conclusion

Our intervention did not improve BP control, despite its success in increasing the number of patients with hypertension attending the clinic, as well as the number that attended on their appointed day. We believe that the study was compromised by the large and increasing demands on primary care, the dominance of the vertically funded HIV programme and the poor standards of equipment in clinics. However, as this study shows, adding additional human resources (even if readily available and relatively inexpensive) is unlikely to have an effect on health outcomes, without the necessary equipment to accurately measure BP, and sufficient clinical staff to treat the growing numbers of chronic patients. To be successful, task shifting interventions need to take account of all aspects of the patient encounter, and if possible other system-wide contextual changes (such as rapidly increasing patients). Our results, taken together with the existing evidence, suggest that LHWs can play an important role in supporting the management of hypertension.
