# Supplementary material for: Can lay health workers support the management of hypertension? Findings of a cluster randomised trial in South Africa
Source: BMJ Glob Health. 2018 Feb 15;3(1):e000577. doi: 10.1136/bmjgh-2017-000577 (PMC5841534; doi:10.1136/bmjgh-2017-000577)
Supplement: Supplementary file 1 [file bmjgh-2017-000577supp001.pdf]

Supplementary File

Table S1 Relationship between usual clinic and last clinic visited

	Usual clinic is Control %(n)				Usual clinic is Intervention %(n)			
Last clinic visited	Baseline		End of intervention		Baseline		End of intervention	
Control	97.7	(1833)	96.6	1312)	2.2	(33)	2.9	(30)
Intervention	2.3	(43)	3.4	(46)	97.8	(1437)	97.1	(1019)
Total	100.0	(1876)	100.0	(1355)	100.0	(1470)	100.0	(1049)
